# Differences in selectivity to natural images in early visual areas (V1–V3)

**DOI:** 10.1038/s41598-017-02569-4

**Published:** 2017-05-26

**Authors:** David D. Coggan, Luke A. Allen, Oliver R. H. Farrar, Andre D. Gouws, Antony B. Morland, Daniel H. Baker, Timothy J. Andrews

**Affiliations:** Department of Psychology and York Neuroimaging Centre, University of York, York, YO10 5DD United Kingdom

## Abstract

High-level regions of the ventral visual pathway respond more to intact objects compared to scrambled objects. The aim of this study was to determine if this selectivity for objects emerges at an earlier stage of processing. Visual areas (V1–V3) were defined for each participant using retinotopic mapping. Participants then viewed intact and scrambled images from different object categories (bottle, chair, face, house, shoe) while neural responses were measured using fMRI. Our rationale for using scrambled images is that they contain the same low-level properties as the intact objects, but lack the higher-order combinations of features that are characteristic of natural images. Neural responses were higher for scrambled than intact images in all regions. However, the difference between intact and scrambled images was smaller in V3 compared to V1 and V2. Next, we measured the spatial patterns of response to intact and scrambled images from different object categories. We found higher within-category compared to between category correlations for both intact and scrambled images demonstrating distinct patterns of response. Spatial patterns of response were more distinct for intact compared to scrambled images in V3, but not in V1 or V2. These findings demonstrate the emergence of selectivity to natural images in V3.

## Introduction

Visual areas involved in object perception form a ventral processing pathway that projects from the occipital toward the temporal lobe. Physiological studies have shown that neurons in early visual areas show selectivity for low-level visual properties such as orientation, spatial frequency and retinal position^1^. Information from early visual areas eventually reaches high-level visual cortex in the temporal lobe, where neurons are tuned to combinations of low-level visual features^2, 3^. Indeed, neurons in different regions of high-level visual cortex have properties that are selective for different categories of objects^4^. For example, some regions respond more to images of faces^5–8^. Other regions show selectivity for images of places^9^, body parts^10^ and visually presented words^11^. Other studies have shown that the spatial pattern of response in these regions is able to discriminate a larger range of object categories^12, 13^.

A characteristic of these high-level regions is their selectivity for intact images^14–16^. At early stages of processing (V1), there are greater responses to scrambled compared to intact images^15^. In contrast, the responses in high-level visual cortex are greater for intact compared to scrambled images. The selectivity for intact images is also evident in the spatial pattern of response of high-level regions of the ventral pathway. More distinct patterns of neural response (defined by higher within- compared to between-category correlations) are found to intact compared to scrambled images^17^. An important feature of intact images is the strong statistical dependencies between features, such as location-specific combinations of orientation and spatial frequency. Indeed, the behavioural sensitivity to the regularities that occur in intact objects suggests that these properties are critical for differentiating between different classes of images^18–22^.

The aim of this study is to determine at what stage these statistical properties of intact objects emerge in the ventral visual pathway. Recent studies have shown that neurons in V2, but not in V1, respond selectively to synthetic textures that are based on the higher-order statistical properties found in natural images^23, 24^. Patterns of response in V2 are better able discriminate these naturalistic textures than control textures that are not based on natural images^25^. In the current study, we compare the response to images of objects and to scrambled versions of objects in early visual areas (V1–V3). Our aim was to determine whether these early visual areas showed selectivity to the statistical properties found in natural images. First, we asked whether the magnitude of response in these regions was greater for intact compared to scrambled images. Second, we asked whether the spatial pattern of response was more distinct for intact compared to scrambled images. Our hypothesis was that, if neurons in early visual areas are selective for statistical regularities found in intact objects, there should be either a greater response or a more distinct pattern of response to intact compared to scrambled images.

## Materials and Methods

### Stimuli

**Figure 1:**
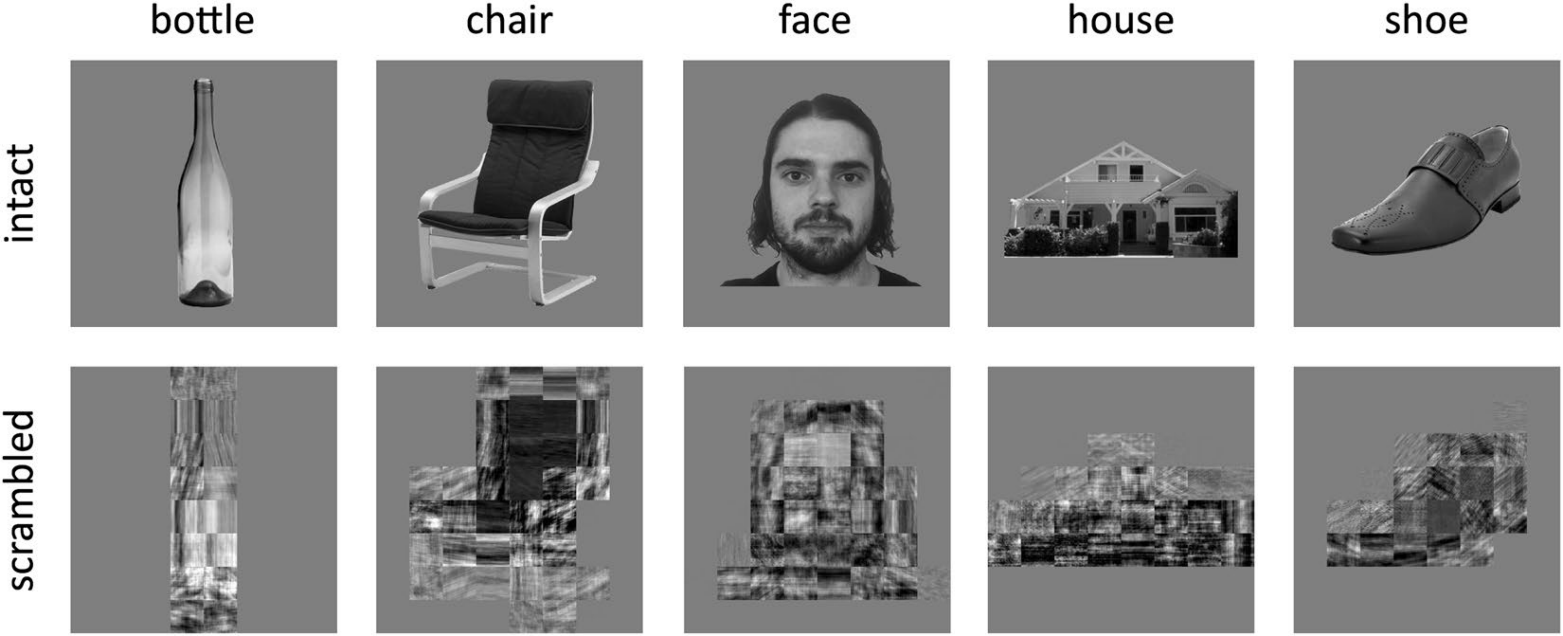
Exemplars of intact and scrambled images from the different object categories.

180 images of five object categories (bottle, chair, face, house, shoe) were taken from an object image stimulus set^26^. All images were gray-scale, superimposed on a mid-gray background, and had a resolution of 680 × 680 pixels. Faces were taken from the Radboud face database (http://www.socsci.ru.nl:8180/RaFD2/RaFD?p=main), which can be used freely for non-commercial scientific research. A scrambled version of each image was created by applying a Fourier phase-scramble to different spatial regions of the image^17^. This involved windowing each image into an 8 × 8 grid and phase-scrambling the contents of each window independently. This process preserves the spatial extent of the images. It also preserves the overall spatial frequency and orientation information (amplitude spectrum). However, the phase scrambling disrupts the specific combinations of image properties that are characteristic and perhaps diagnostic of particular semantic categories. Images subtended a maximum retinal angle of approximately 15° and were viewed on a screen at the rear of the scanner via a mirror placed immediately above the participant’s head. Examples of the stimuli are shown in Fig. 1.

### Participants

Twenty-one participants took part in the fMRI experiment (10 male, mean age = 26.3, SD = 6.0 years). All participants had normal or corrected-to-normal vision. Each gave their informed, written consent and the study was approved by the York Neuroimaging Centre (YNiC) Ethics Committee and adhered to the Declaration of Helsinki.

### Design and Procedure

There were 10 conditions: 5 categories (bottle, chair, face, house, shoe) × 2 image types (intact, scrambled). Images were presented in 6 s blocks. In each block, 6 images from a condition were presented individually for 800 ms, with a 200 ms inter-stimulus-interval. This was followed by a fixation cross lasting 9 s. There were 6 repetitions of each condition in the scan. To maintain attention participants were instructed to press a button on a response box whenever a red dot appeared on an image, which occurred once in each block. On average, subjects responded to 99.3% (SEM = 0.04%) of red dot images, with a mean reaction time of 420 msec (SEM = 14 msec). There was no significant difference in the number of hits between intact (mean = 99.4%, SEM = 0.3%) and scrambled (mean = 99.2%, SEM = 0.5%) conditions (t(20) = 0.37, ns). There was also no significant difference in the response latencies between intact (mean = 417 msec, SEM = 15 msec) and scrambled (mean = 425 msec, SEM = 14 msec) conditions (t(20) = 1.56, ns).

### Data Acquisition

fMRI data were acquired with a General Electric 3 T HD Excite MRI scanner at YNiC at the University of York, fitted with an eight-channel, phased-array, head coil. A gradient-echo echo-planar imaging (EPI) sequence was used to collect data from 38 contiguous axial slices (TR = 3000 ms, TE = 32.7 ms, FOV = 288 × 288 mm, matrix size = 128 × 128, slice thickness = 3 mm). The fMRI data were initially analyzed with FEAT v5.98 (http://www.fmrib.ox.ac.uk/fsl). In all scans, the initial 9 s of data was removed to reduce the effects of magnetic saturation. Motion correction (MCFLIRT, FSL) and slice-timing correction were applied followed by temporal high-pass filtering (Gaussian-weighted least-squares straight line fitting, sigma = 50 s). Gaussian spatial smoothing was applied at 6 mm FWHM.

### Region of Interest Localization

**Figure 2:**
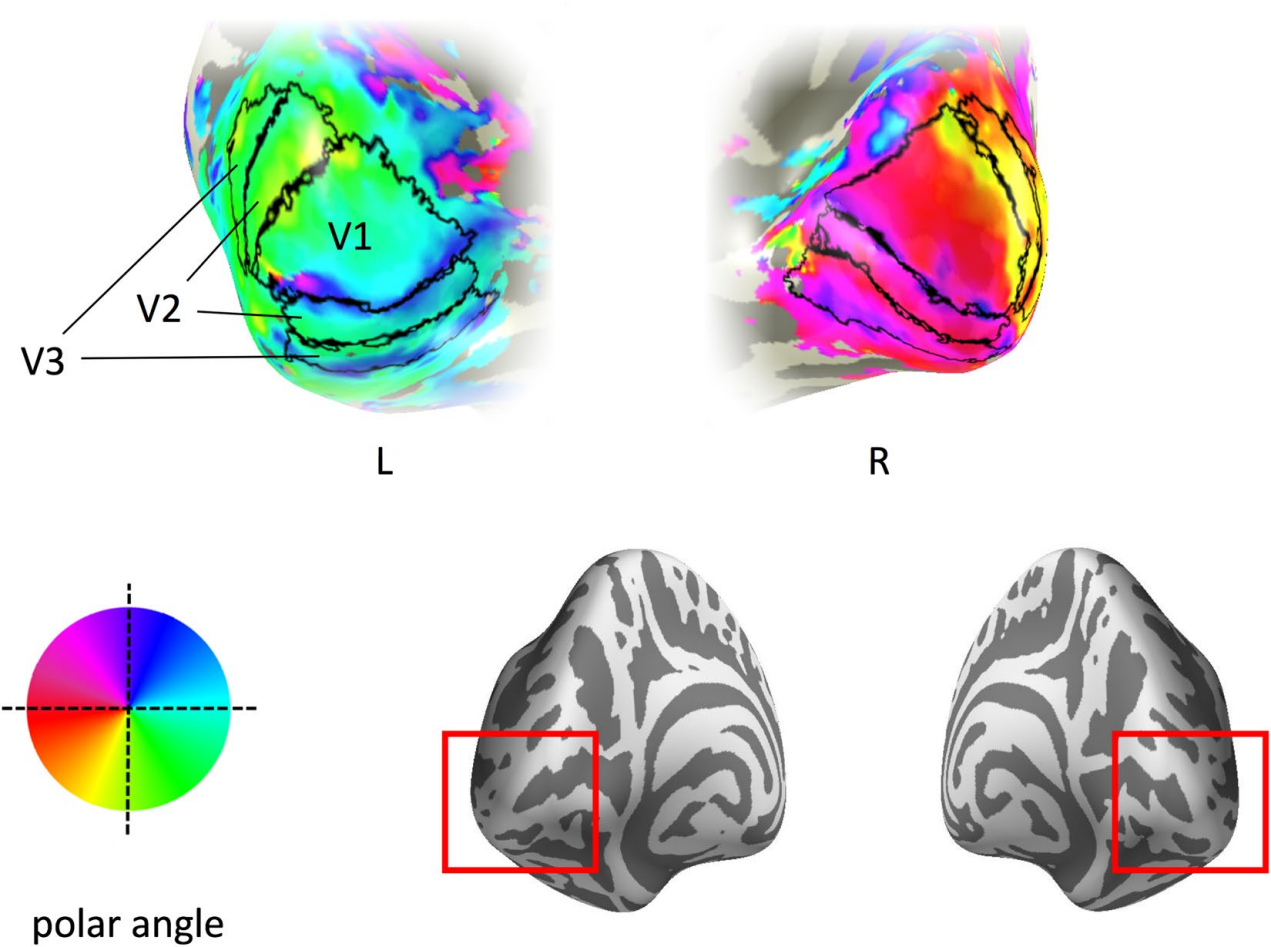
Early visual cortical regions for a representative participant. Visual areas are superimposed onto the occipital lobe – see red insert on the posterior view of the inflated brain. Colour maps indicate the preferred polar angle.

Visual areas were defined in a separate scan session (TR, 3000 ms; TE, 30 ms; voxel size, 2 × 2 × 2 mm^3^; flip angle, 90°; matrix size, 96 × 96 × 39; FOV, 19.2 cm) with a 16-channel head coil to improve signal-to-noise in the occipital lobe using either ring and wedge type stimuli^27^ or population receptive field techniques^28^. Wedges rotated counterclockwise about a red fixation cross. Ring stimuli expanded about fixation. Both wedges and rings were high contrast checkerboard stimuli that flickered at a rate of 6 Hz. Each scan contained eight cycles of wedges/rings, with 36 s per cycle, traversing a circular region of radius 14.3°. Participants maintained fixation throughout the scan. Visual area boundaries between V1/V2 and V2/V3 (dorsal and ventral) were defined by the phase reversals in the polar angle representations on inflated representations of the visual cortex (Fig. 2, Supplementary Figure 1). Visual field eccentricity representations were used to restrict the ROI to the location of the stimulus, i.e. the central 15° of visual angle. Functional data from the main experimental scan were aligned to a high-resolution T1-anatomical image that was segmented into gray matter and white matter.

### Data Analysis

To compare the magnitude of response to different stimulus conditions, parameter estimates were generated for each condition by regressing the hemodynamic response of each voxel against a boxcar function convolved with a single-gamma HRF. The responses from each voxel were then averaged within each ROI and converted from units of image intensity to % signal change. A repeated measures ANOVA was then used to determine the effect of ROI (V1, V2, V3) and Image Type (intact, scrambled).

To compare the spatial patterns of neural response, parameter estimates were generated for odd and even runs of each condition by regressing the hemodynamic response of each voxel against a box-car function convolved with a single-gamma HRF. Parameter estimates were normalized by subtracting the mean response per voxel across all conditions (odd and even, intact and scrambled). These data were then submitted to a within-subjects, correlation-based multivariate pattern analysis^12, 29^ (MVPA) implemented using the PyMVPA toolbox^30^ (http://www.pymvpa.org/). This allowed us to compare spatial patterns of response to all combinations of objects. For within-category comparisons, the correlation between responses in odd and even runs was used. For between-category comparisons, the mean correlation across odd-even and even-odd contrasts was used. A Fisher’s Z-transformation was then applied to the correlations prior to further statistical analyses. If there are distinct patterns for each object category, there should be a higher correlation in the spatial pattern of response for within-category compared to between-category comparisons.

## Results

**Figure 3:**
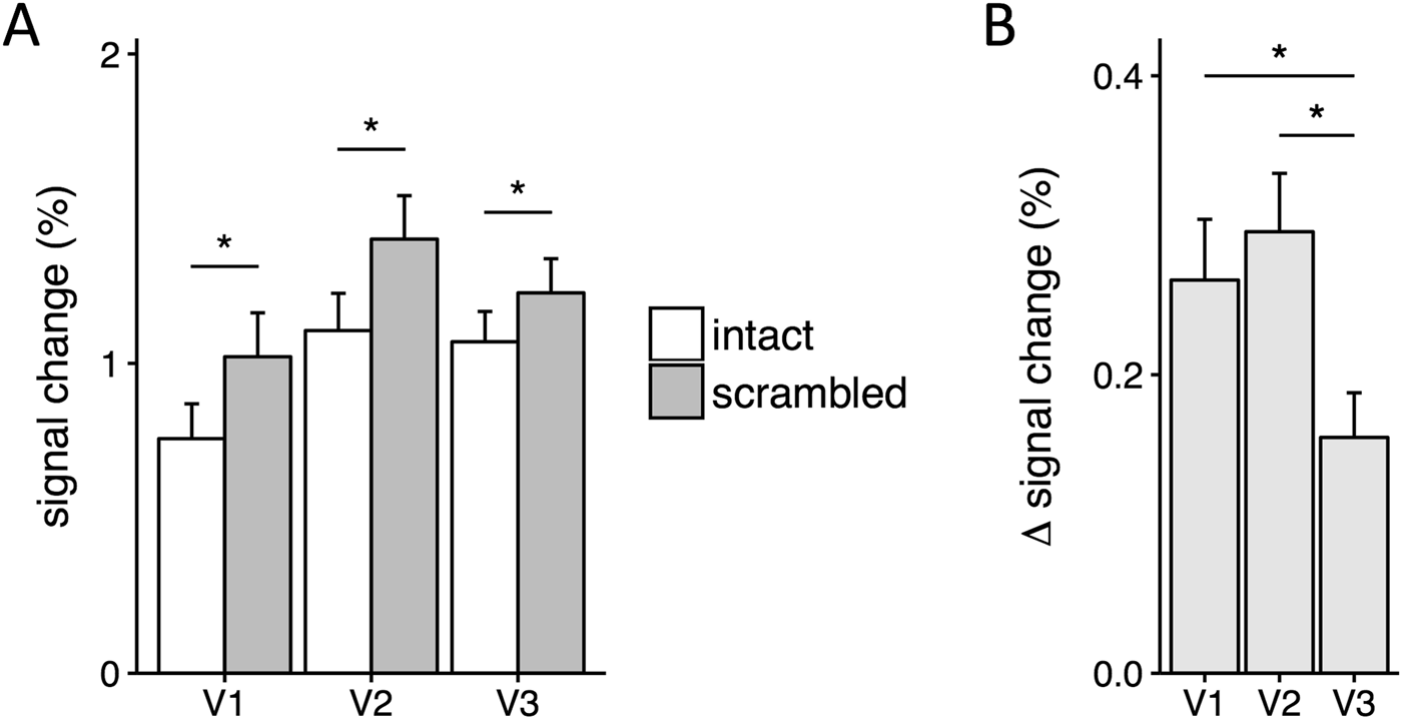
(**A**) Magnitude of response to intact and scrambled images. Scrambled images evoked more activity than intact images in each visual area. (**B**) Differences in response to intact and scrambled images for each visual region. V3 showed a smaller difference in response to intact and scrambled images compared to V1 and V2. Error bars show ±1 SEM. *p < 0.05, FDR corrected.

First, we asked whether the overall neural response in V1, V2 and V3 could distinguish intact and scrambled images. To address this question, we measured the % signal change in each region to intact and scrambled images (Fig. 3). We then performed a 2-way ANOVA with Region (V1, V2, V3) and Image Type (intact, scrambled) as factors. There was a significant main effect of Image Type (F(1,20) = 54.67, p < 0.0001) and a significant interaction between Region and Image Type (F(2,40) = 10.83, p = 0.0002). Pairwise comparisons revealed that scrambled images evoked more activity than intact images in V1 (t(20) = 6.46, p < 0.0001), V2 (t(20) = 7.55, p < 0.0001) and V3 (t(20) = 5.28, p = 0.0001). However, this difference was significantly smaller in V3 compared to both V1 (t(20) = 3.13, p = 0.0079) and V2 (t(20) = 5.54, p < 0.0001) (see Fig. 3B). This is the cause of the interaction detected by the ANOVA analysis as there was no difference between V1 and V2 (t(20) = 0.97, ns).

**Figure 4:**
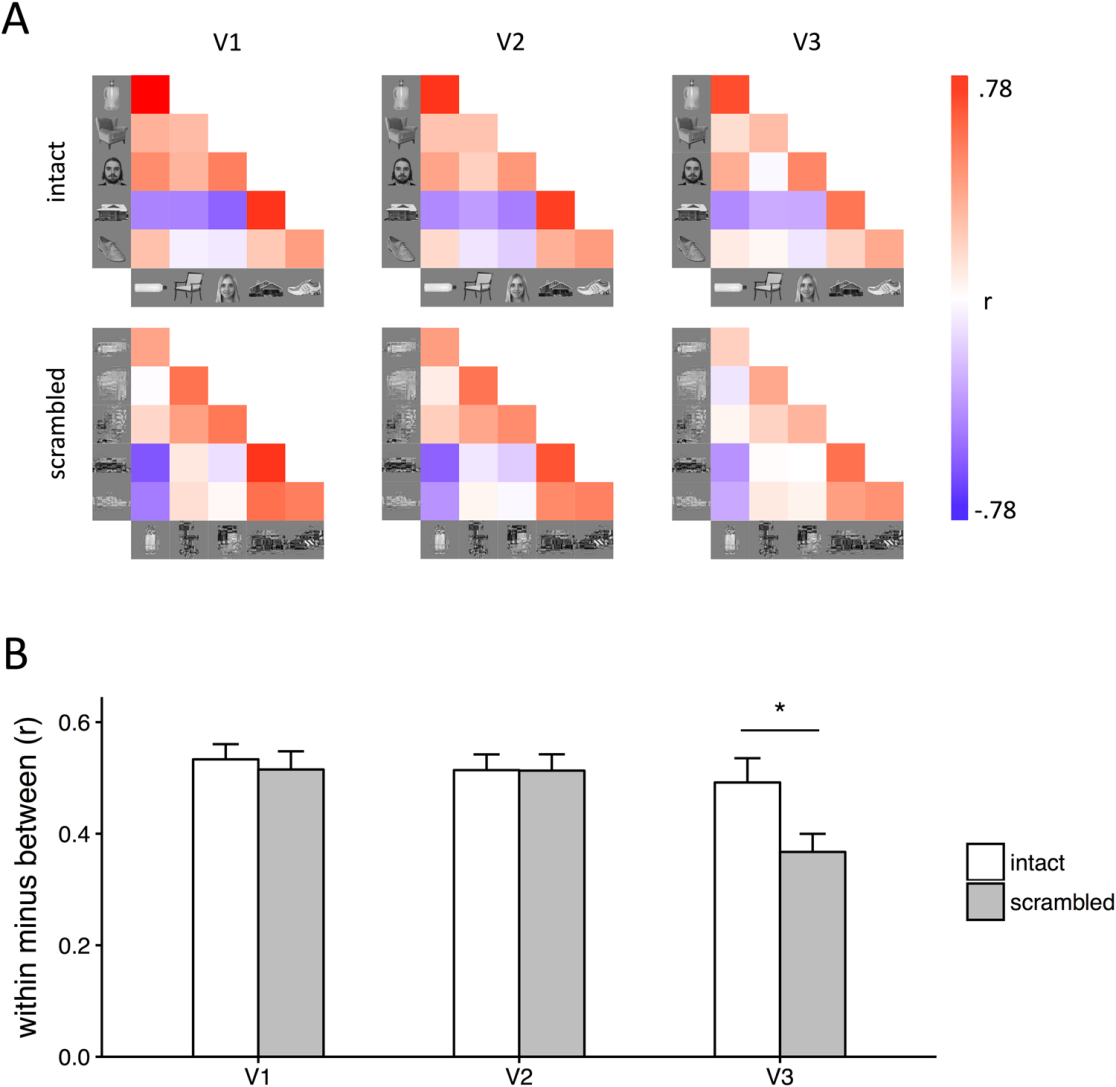
(**A**) Similarity matrices showing the correlation in patterns of neural response to all within-category and between-category comparisons. Within-category comparisons (e.g. bottle-bottle) are shown on the diagonal. (**B**) Bar graph showing the mean within-category and between-category correlations for intact and scrambled images across participants. There was a significant interaction between Comparison, Image Type and Region, which was due to more distinct (within > between) patterns of neural response to intact relative to scrambled images in V3. Error bars show ±1 SEM. *p < 0.05, FDR corrected.

Next, we asked whether there were differences in the spatial patterns of response in V1, V2 and V3 to intact and scrambled images. To address this question, we first tested whether different intact and scrambled object categories evoked distinct and reliable patterns of fMRI response in regions V1, V2 and V3 (Fig. 4 and Supplementary Figure 2). We compared the similarity of patterns of response to images from the same category (e.g. bottle vs. bottle) with the similarity of patterns to images of different categories (e.g. bottle vs. chair). Distinct, category-specific patterns of response are indicated by the within-category correlations being significantly greater than the between-category correlations (see Fig. 4b).

A 3-way repeated-measures ANOVA was performed with Comparison (within-category, between-category), Region (V1, V2, V3) and Image Type (intact, scrambled) as factors. There were main effects of Comparison (F(1,20) = 383.15, p < 0.0001) and Region (F(2,40) = 10.40, p = 0.0002). Although there was no effect of Image Type (F(1,20) = 1.24, p = 0.28), there was a significant three-way interaction between Comparison, Region and Image Type (F(2,40) = 5.28, p = 0.0093). This indicated that the distinctiveness of the category-specific patterns of response reflected by the effect of Comparison (within-category - between-category) differed across intact and scrambled images, depending on the visual region. Pairwise comparisons revealed that intact images evoked more distinct category-specific patterns than scrambled images in V3 (t(20) = 2.99, p = 0.020). This difference in the spatial pattern of response was not seen in V1 (t(20) = 1.17, ns) or V2 (t(20) = 0.71, ns). This shows that the spatial pattern of response to different object categories is more distinct for intact compared to scrambled images in V3.

**Figure 5:**
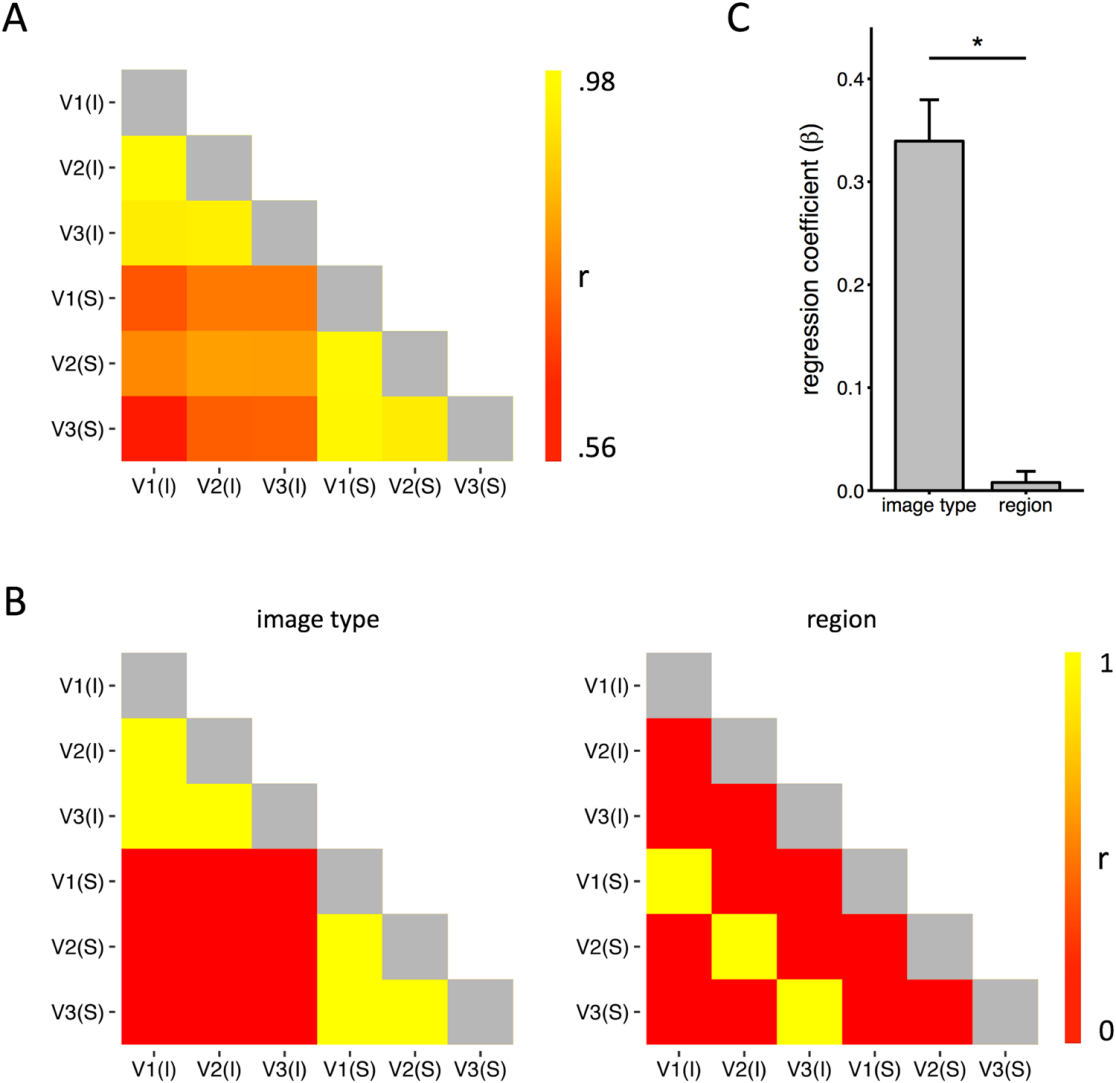
(**A**) Correlations between the similarity matrices shown in Fig. 4A. (**B**) Matrix predictions based on representations of image type and region. (**C**) Models were used in a regression analysis across participants. Performance was determined by the regression coefficients for each model. The results show that patterns of response were predicted significantly more by the image type than region. Error bars show ±1 SEM. *p < 0.05.

Finally, we investigated the patterns of response similarity to different object categories across image type and region. Figure 5A shows all pairwise correlations across the different similarity matrices shown in Fig. 4. This appears to show higher correlations to the same image type (intact or scrambled). For example, in V1 the correlation with V2 and V3 for intact images was 0.99 and 0.96, respectively. In contrast, the correlation between intact and scrambled images in V1 was 0.63. To assess whether the higher correlation for the same image type was statistically significant, we generated two models: Image Type and Region (5B). These models were regressed onto each subject’s matrix, generating a distribution of beta-weights for each model (5C). These weights were significantly above zero for Image Type (t(20) = 8.45, p < 0.0001), but not for Region (t(20) = 0.73, ns). Weights for Image Type were also significantly higher than those for Region (t(20) = 8.26, p < 0.0001). This shows that representational distances between the object categories across all visual areas was different for intact and scrambled images.

## Discussion

The aim of this study was to determine whether early stages of the ventral visual pathway are selective for objects. To address this issue, we compared both the magnitude and the pattern of response to intact and scrambled images from different object categories in V1, V2 and V3. Our results reveal that all regions showed greater overall neural response to scrambled images relative to intact images. However, this difference was smaller in V3 compared to V1 and V2. We also found that the spatial pattern of response in V3, but not in V1 or V2, was more distinct for intact objects compared to scrambled objects. Our results show that this selectivity for the properties of natural objects begins to emerge in the response properties of V3.

The majority of studies of the human object-recognition pathway have focused on the initial (V1) or the final (category-selective regions) stages of processing, while the intermediate stages have received less attention^31^. Neurons in V1 are known to be selective for low-level features of the image^1^. Further downstream, neurons are tuned to properties that appear to combine features encoded in earlier visual areas that are statistically characteristic of natural images^2–4, 29^. To determine where selectivity emerges, we compared responses to intact and scrambled objects in early visual areas (V1–V3). Scrambled images contain the same visual elements as intact images, but lack the statistical regularities between elements that are important for object perception^20–22^. Previous studies have shown differential responses to intact and scrambled objects along the ventral visual pathway^14, 15^. For example, Grill-Spector and colleagues used a box-scrambling method to progressively change the degree of scrambling. In early visual areas (V1–V3), they found a higher response to all the scrambled conditions compared to intact images. V4 showed a maximal response to intermediate levels of scrambling and higher visual areas responded most strongly to intact images. These findings suggest that V4 is an important intermediate region in the neural representation of objects^32–34^. However, previous studies have either not reported the responses in V3 or have not distinguished between the response properties of V3 and V1/V2. We found that all regions (V1–V3) showed higher responses to scrambled compared to intact images, but that this difference was attenuated in V3. This suggests that some of the selectivity in V4 to higher order properties of the image may emerge from V3.

To further probe the selectivity to objects in V3, we compared the spatial pattern of response to intact and scrambled images. This analysis is based on a comparison of the within-category similarity in spatial response with the between-category similarity. In a recent study, we found that category-selective patterns of response in high-level regions of the ventral pathway to scrambled images are less distinct than for intact images^17^. We found that the distinctiveness of the pattern of response to different object categories in V3 was greater for intact compared to scrambled images. This reliability in the spatial pattern of response to intact objects is consistent with other studies that have found that temporal patterns of neural response are also more reliable with natural images^35^. These findings complement the univariate analysis and show that a bias toward natural images begins at an early stage of processing. It is important to note, however, that these findings only show the emergence of selectivity rather than the full selectivity found in higher visual areas. In our opinion, it is likely that selectivity for objects is an emergent property, rather than a binary property, of the visual system.

Although neurons in V2 receive most of their input from V1 and have similar selectivity for orientation and spatial scale^36^, a number of studies have shown differences in the response to conjunctions of image features in V2^37–39^. Recent studies have found that neurons in V2 show larger and more reliable responses to synthetic textures that have properties based on natural images compared to control textures^23–25^. Given this sensitivity to the higher order structure of more naturalistic stimuli, the lack of difference between V1 and V2 in our study was unexpected. One possibility is that the objects used in the current study lack the regularity in structure found in the textures generated by Simoncelli and colleagues^40^. Nevertheless, these findings fit with a recent study showing responses to different texture patterns could be differentiated in V3, but not in V2^41^.

Our final analysis involved comparing the representational distances between object categories for intact and scrambled images. Despite the fact that the low-level features were matched between the intact and scrambled images, the representational similarity was more similar for the same image type (intact or scrambled) across regions than for different image types within the same region. For example, the similarity in the representational similarity between V1 and V2 for intact images was greater that the representational similarity between intact and scrambled images in V1. This suggests early visual areas are sensitive to the statistical regularities found in intact natural images.

Anatomical observations have shown that neuronal density decreases along the posterior-anterior axis of the primate visual system^42^. This is accompanied by a corresponding reduction in the surface area of regions in higher visual areas^43^. Taken together, these findings indicate that there is a reduction in the amount of information encoded at higher levels of the ventral stream^44^. This places constraints on the number of feature conjunctions that can encoded^45^. One solution to this combinatorial problem is to only encode combinations of low-level features that are commonly found in natural objects^46, 47^. We found that the difference between the patterns of response to intact and scrambled images was a reduction in the distinctiveness of the scrambled images. This suggests that the adaptive encoding that is necessary for successful object perception begins at an early stage of processing.

In conclusion, the ventral visual pathway comprises a sequence of cortical areas in which successively more complex visual attributes are extracted, beginning with contour orientations in V1 and resulting in representations of objects at the highest levels. In contrast, high-level regions of the ventral visual pathway produce greater or more reliable responses to natural, intact images relative to artificial, scrambled images. Previous studies have been unclear at which stage in the processing stream this selectivity to natural images emerges. Here, we show a preference for natural images can be found at early stages of processing in extrastriate visual cortex.

## Electronic supplementary material

Supplementary Dataset
