# Supplementary material for: Differences in selectivity to natural images in early visual areas (V1–V3)
Source: Sci Rep. 2017 May 26;7:2444. doi: 10.1038/s41598-017-02569-4 (PMC5446401; doi:10.1038/s41598-017-02569-4)
Supplement: Supplementary file 1 — Supplementary Dataset [file 41598_2017_2569_MOESM1_ESM.doc]

**DIFFERENCES IN SELECTIVITY TO NATURAL IMAGES IN EARLY VISUAL AREAS (V1- V3)**

**(SUPPLEMENTARY INFORMATION)**

David D. Coggan, Luke A. Allen, Oliver R.H. Farrar, Andre D. Gouws, Antony B. Morland, Daniel H. Baker & Timothy J Andrews*

Department of Psychology and York Neuroimaging Centre,

University of York, York, YO10 5DD, United Kingdom

Corresponding author: timothy.andrews@york.ac.uk

**
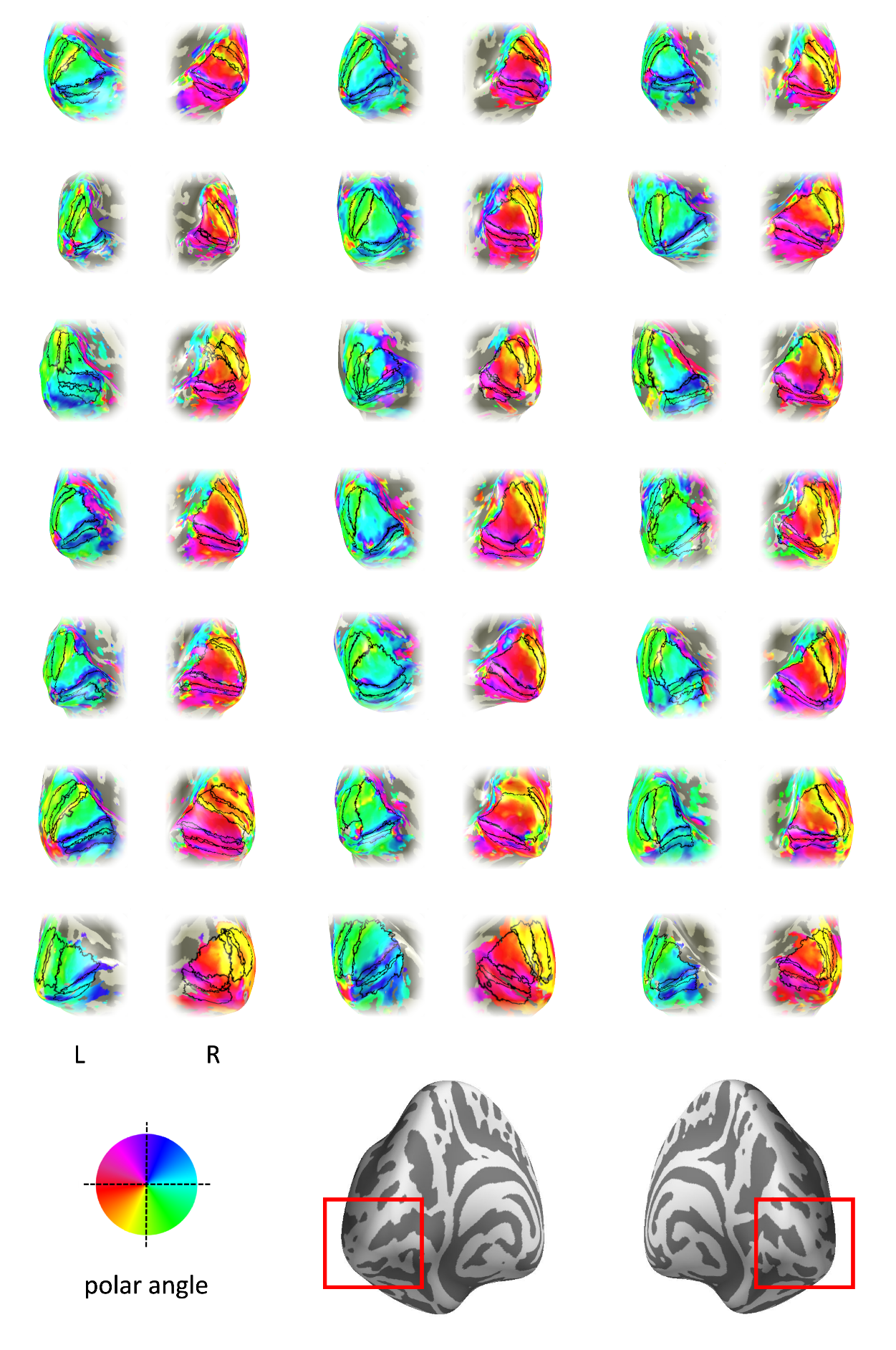
**

**Supplementary Figure 1** Early visual cortical regions for all participants. Visual areas are superimposed onto the occipital lobe – see red insert on the posterior view of the inflated brain. Colour maps indicate the preferred polar angle.


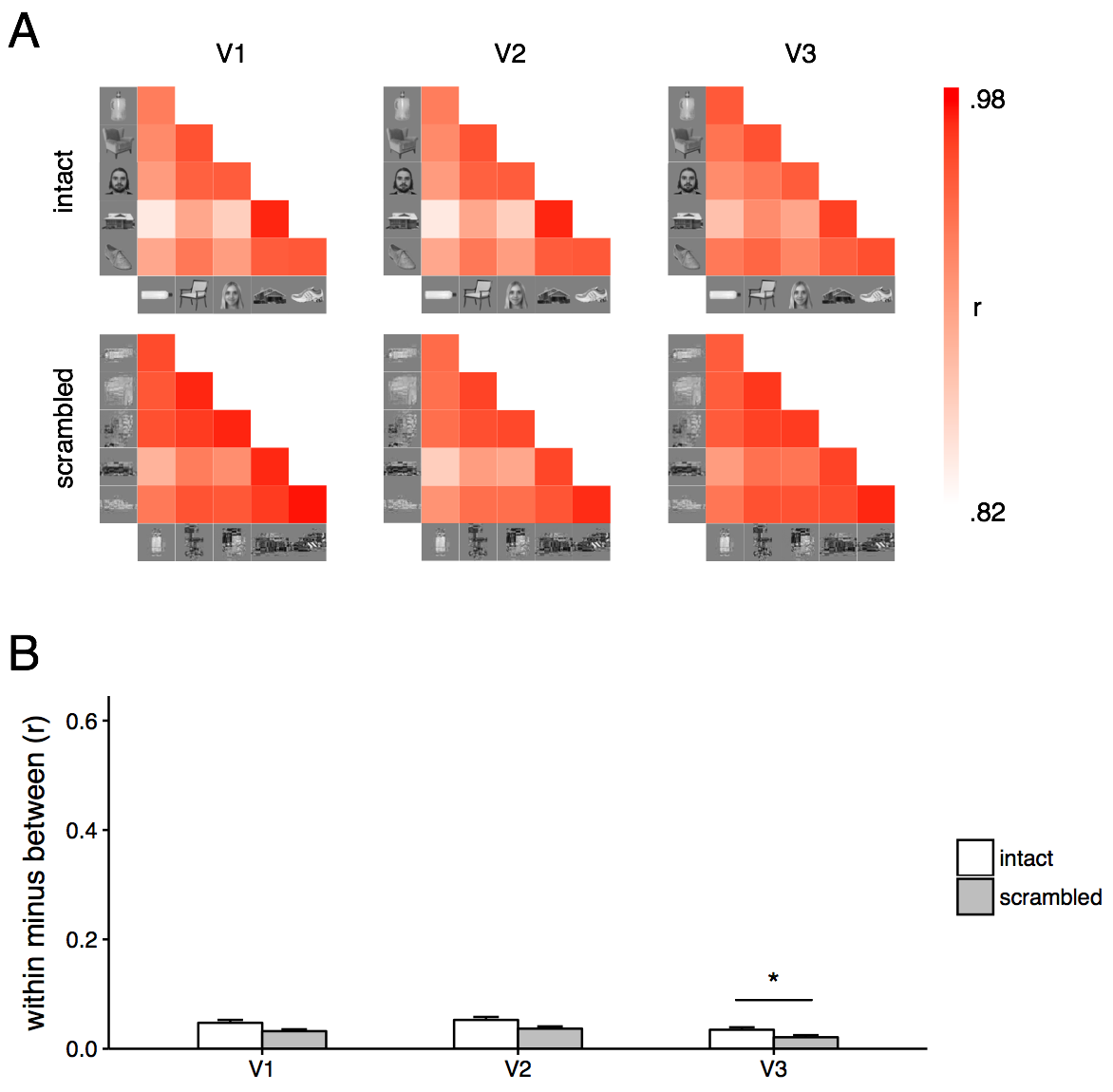


**Supplementary Figure 2** MVPA on unnormalized data. (A) Similarity matrices showing the correlation in patterns of neural response to all within-category and between-category comparisons. Within-category comparisons (e.g. bottle-bottle) are shown on the diagonal. (B) Bar graph showing the mean within-category and between-category correlations for intact and scrambled images across participants. A 3-way repeated-measures ANOVA was performed on the z-transformed r values with Comparison (within-category, between-category), Region (V1, V2, V3) and Image Type (intact, scrambled) as factors.  There were main effects of Comparison (F(1,20) = 368.65, p < .0001), Region (F(2,40) = 4.68, p = .0149) and Image Type (F(1,20) = 69.74, p = <.0001).  There was a significant three-way interaction between Comparison, Region and Image Type (F(2,40) = 6.88, p = .0027).  This indicated that the distinctiveness of the category-specific patterns of response reflected by the effect of Comparison (within-category - between-category) differed across intact and scrambled images, depending on the visual region.  Pairwise comparisons revealed that intact images evoked more distinct category-specific patterns than scrambled images in V3 (t(20) = 2.49, p = .0217).  This difference in the spatial pattern of response was not seen in V1 (t(20) = 0.27, ns) or V2 (t(20) = 0.69, ns). This shows that the spatial pattern of response to different object categories is more distinct for intact compared to scrambled images in V3. Error bars show ±1 SEM. * p < .05, FDR corrected.
